# Patient Safety Incidents Caused by Poor Quality Surgical Instruments

**DOI:** 10.7759/cureus.4877

**Published:** 2019-06-10

**Authors:** Elizabeth D. Dominguez, Brett Rocos

**Affiliations:** 1 Orthopaedics, University of Bristol Medical School, Bristol, GBR; 2 Orthopaedics, North Bristol National Health Service Trust, Bristol, GBR

**Keywords:** patient safety, surgical instruments, drillbits, quality, surgery

## Abstract

Objectives: Surgeons require high-quality surgical instruments to carry out successful procedures. Poor quality instruments may break intraoperatively leading to a failed procedure or causing harm to the patient. By examining the National Reporting and Learning Service (NRLS) database, the study aims to define the scale of the problem and provide evidence for the formation of surgical instrument quality control.

Methods: The NRLS was searched from August 2004 - December 2010. The search revealed 2036 incidents, 250 of which were randomly selected and analyzed by a clinical reviewer.

Results: One hundred and sixty-one incidents were identified causing five reoperations, one incident of severe harm, six incidents of moderate harm, 35 of low harm, and 119 no harm incidents. No patient deaths were discovered. Drillbits were the most commonly broken instrument.

Conclusions: This report is likely to only be the tip of the iceberg. Poor reporting of patient safety incidents means that there may be as many as 1500 incidents a year of poor quality surgical instruments causing harm. We suggest that forming a Surgical Instrument Quality Service at Trusts within the National Health Service (NHS) could prevent harm coming to patients, reduce cost, and improve the outcomes of surgical procedures.

## Introduction

The quality of surgical instruments is heavily relied upon by surgeons to perform procedures to the highest standard. Purchased from reputable suppliers, it is presumed that these instruments are of high quality and are usually put to use before any final user quality control is carried out. Problems arise, however, when these instruments do not live up to these expectations. Poor quality instruments fail and break and when used in an operation, the consequences to the patient can be disastrous. In the United States, the Food and Drug Administration (FDA) published an alert in 2008 stating that nearly 1000 incidents of retained pieces of broken instruments (unretrieved device fragments, UDFs) occurred each year, leading to a range of problems including local tissue reactions, infections, disability, and even death [1]. The alert also notes that with the increasing use of magnetic imaging modalities, the unrecognized presence of ferrous foreign bodies may cause tissue trauma or thermal injury.

In the United Kingdom, Daly et al. reported on a pilot study of a Surgical Instruments Service to assess the quality of instruments purchased by the hospital, remove those unfit for purpose, and inform the manufacturer [2]. Brophy et al. found that 15% of surgical instruments examined by medical and mechanical engineers failed to meet the appropriate British Standards (BS) guidelines [3]. Instruments purchased from certain manufacturers failed at a rate of 35%, suggesting that potentially one in every three instruments in some sets are of substandard quality [4].

In this study, we aim to define and examine the problem of UDFs by observing the contribution of poor quality surgical instruments to reports of patient safety incidents made to the National Reporting and Learning System (NRLS) of the National Health Service (NHS) in England and Wales. In doing so, evidence for the requirement of surgical instrument quality control can be determined.

## Materials and methods

**Figure 1:**
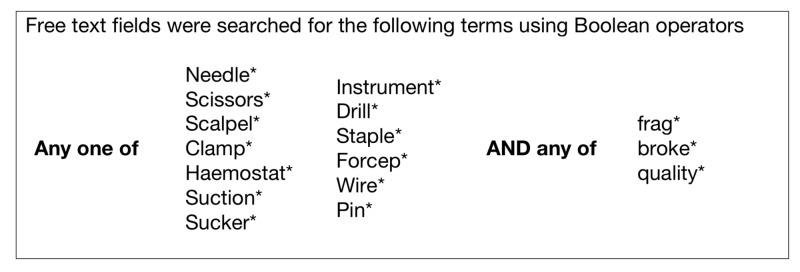
The terms used to search the National Reporting and Learning System database. The asterisk (*) is a truncation or 'wild card' search operator. It is attached to a search term so that when the search strategy is run, other forms of the search term will also be matched. In this case, any word will match if it begins with the word preceded by the asterisk (*).

The NRLS database was searched using the terms in Figure 1 for the period of August 2004 - December 2010. This initial search produced 2036 safety incidents, of which 250 were randomly selected using computer software (SAS 4.2, using the Proc Survey feature; SAS Institute Inc. Cary, NC, USA). These results were then screened by one Clinical Reviewer at the National Patient Safety Agency (NPSA) for relevance to the question asked. Records that were misfiled by the NRLS were eliminated from the analysis, as were records where instrument breakage was due to human error, e.g., dropping the instrument on the floor. All remaining records were included.

## Results

Of the 250 case reports randomly selected from the NRLS system, 161 were relevant to this study. This equates to 64.4%, suggesting that the overall number of relevant incidents reported to the database as a whole is somewhere in the region of 1310 patient safety incidents due to faulty instruments across five years (approximately 260 cases per year).

One of the obligatory fields in the patient safety incident report is the degree of harm. In the incidents analyzed for this study, the degree of harm is detailed in Table 1.

**Table 1:** The degree of harm caused by potentially poor-quality instruments reported to the National Reporting and Learning System.

No harm	Low harm	Moderate harm	Severe harm	Total
119	35	6	1	161

**Figure 2:**
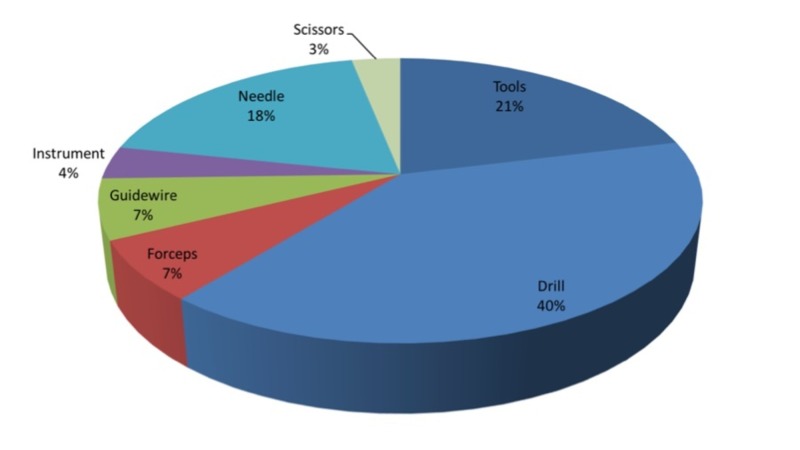
Distribution of broken instruments in reported patient safety incidents between August 2004 and December 2010.

Based on Figure 2, it is immediately obvious that broken drill bits constitute the largest group of broken instruments (40%). The category ‘instruments’ comprises broken instruments not adequately detailed in the incident report; the group labeled ‘tools’ is further described in Table 2.

**Table 2:** Breakdown of broken instruments included in the 'Tools' category

Instrument	Frequency
Arthroscopy instrument	1
Depth gauge	1
McDonalds	1
Screwdriver	1
Shears	1
Ventouse device	1
Blade	2
Diathermy instrument	2
Pin	2
Saw	2
Stone crusher	2
Suction catheter	2
Clamp	3
Snare	3
Retractor	4
Burr	5

Of the 161 included incidents, unintended metal was left in 77 patients, broken fragments were removed in only 57 cases but most alarmingly, 27 reports did not state whether pieces had been removed or not or did not mention whether X-ray assessment had taken place.

Five records showed that further operations were required to remove UDFs. Along with the concomitant risks that these operations carried were the additional risks associated with UDF removal.

## Discussion

UDFs pose a serious problem if the issue remains unattended. If the results of the above analysis are generalized, there may be as many as 262 faulty instruments related to patient safety incidents reported to the NPSA each year. Although no deaths appear to have occurred in this sample, serious harm was brought to 1 patient and 5 went on to have further operations to remove the foreign object. Such reoperations incur additional associated risks and costs of emergency procedures which may bring about serious harm. These are certainly adverse outcomes that could be avoided altogether.

Although the FDA article released in 2008 [1] discusses a wide range of untoward effects of UDFs, Pichler et al. showed that although 37 UDFs were reported following nearly 12,000 orthopedic cases in two hospitals between 2005 and 2006, not a single patient suffered any untoward problems over a 12-36 month follow up period [5]. This, coupled with the reported low harm outcomes, might suggest that UDFs are not a large problem in the United Kingdom and that those that do occur lead to minimal harm. There are, however, some provisos to this conclusion. First and foremost is the nature of the data collected by the NRLS. Given that the reporting of patient safety incidents is voluntary, patient safety incidents are known to be under-reported [6], suggesting that the problem of instrument breakage is likely to be much larger than represented here. Sari et al. looked at ward patients and the occurrence of reporting of patient safety incidents. They found that only 17% of incidents were reported through the proper channels [6]. If this is extrapolated to the situation here, it could be hypothesized that as many as 1500 instrument breakages could be occurring each year, the vast majority of which were not reported to the NRLS. However, it should be mentioned that incidents within the operating theatre usually involve senior staff adhering to a strict set of guidelines, deviation from which may be more likely to stimulate an incident report; it is, therefore, probable that the true number of unreported patient safety incidents is much lower. Nevertheless, uncertainty surrounding the discrepancy between the number of reported incidents versus the true number of incidents highlight the need for improved reporting guidance.

More worrying is perhaps that lack of clarity in many of the reports. UDFs are a potentially serious problem with severe consequences. Many reports do not clarify whether X-ray imaging was used to exclude the presence of a UDF or to confirm whether the pieces had been removed. In addition, several reports have detailed that the responsible clinician deemed an X-ray not necessary. It would seem sensible to carry out a radiological investigation in the event of a suspected UDF to identify the problem and inform prognosis. This not only highlights a limitation of the reporting system which does not require such information in the proforma, but also the need for guidelines in the event of a suspected UDF to document this problem and inform prognosis. The Association for Perioperative Practice guidelines for instrument checks currently states that, "we must ensure that we do not cause any harm to our patients by negligently leaving foreign objects within patient cavities during clinically invasive procedures"; this suggests that some investigation to prove the absence of foreign material, is necessary despite the additional risks involved, although it does go on to state that the use of X-ray for poorly radio-visible items (such as needles) is at the discretion of the surgeon.

If we accept that surgical instruments are very rarely misused so as to exceed their design limits, the incidence of patient injury could be significantly reduced through quality assurance processes of surgical instruments prior to their application. This would help reduce instrument breakages. Additionally, informing the Medicines and Healthcare Products Regulatory Agency (MHRA), the NRLS and the manufacturers themselves of substandard instruments could flag the importance of improving the production quality to limit poor quality instrument distribution.

## Conclusions

Poor-quality control of surgical instruments leads to the use of poor-quality instruments that fail and break. Although there was a low frequency of severe harm observed due to the use of such instruments, their consequences can be disastrous. The alarmingly high frequency of patient safety incidents caused by broken instruments reported to the NRLS per year, in combination with poor reporting of its management warrant the need for preventative and contingency measures in the event of a UDF. The development of surgical instrument quality control and the implementation of guidelines in the event of a suspected UDF should be considered to alleviate the problem.
